# Diversity competence: what should be prioritised in an online course? An adapted delphi study

**DOI:** 10.1093/eurpub/ckac129.731

**Published:** 2022-10-25

**Authors:** J Sorensen, C Michaëlis, JM Møller Olsen, A Krasnik, K Bozorgmehr, S Ziegler

**Affiliations:** 1 Danish Research Centre for Migration, Ethnicity and Health, University of Copenhagen, Copenhagen, Denmark; 2 Department of General Practice and Health Services Research, Heidelberg University Hospital, Heidelberg, Germany; 3 School of Public Health, University of Bielefeld, Bielefeld, Germany

## Abstract

**Background:**

Population diversity is a reality in our societies and requires health systems and health professionals to adapt to the needs of diverse patient groups, including migrants and ethnic minorities. This study aims to investigate topics and methods that should be prioritised in an online course on diversity competence in healthcare delivery to improve health care encounters and provide health services that meet the unique needs of all patients in order to reduce health disparities.

**Methods:**

The study uses an adapted Delphi method including two rounds, combining some open-ended questions with pre-defined items, asking 31 European academic experts and health professionals within the field of migrant health to rate training content and teaching methods. Consensus for training topics was set to 80% and for teaching methods 70%.

**Results:**

The only item reaching 100% consensus as being important or very important to include was ‘health effects of migration (pre-, mid- and post-migration risk factors)’. Other high-scoring items were ‘social determinants of health’ (97%) and ‘discrimination within the healthcare sector’ (also 97%). A general trend was to focus more on reflective practice since almost all items in the reflection section reached consensus. ‘Reflection on own stereotypes and prejudices’ reached the highest consensus in this section (97%).

**Conclusions:**

Experts’ prioritisations of teaching content and methods for diversity training can help the design of short online trainings for health professionals and reduce extensive course content, thereby fostering professional development and enabling diversity competence trainings to be implemented in cases of scarce resources.

**Key messages:**

• Trend toward more focus on ‘diversity’ and less focus on ‘culture, and the inclusion of social determinants of health and awareness of stereotypes and bias in training of health professionals.

• Diversity competence training should use reflective exercises and activities as teaching methods in online training.

